# MuFaDDG: a sequence-based multiscale feature fusion framework for protein stability changes prediction

**DOI:** 10.1093/bioinformatics/btag196

**Published:** 2026-04-29

**Authors:** Jianting Gong, Pengjia Ma, Zilin Ren, Si Li, Zhiguo Fu, Pingping Sun, Ming Ni, Xiaochen Bo

**Affiliations:** Academy of Military Medical Sciences, Beijing, 100850, China; School of Information Science and Technology, Northeast Normal University, Changchun, 130117, China; Academy of Military Medical Sciences, Beijing, 100850, China; School of Information Science and Technology, Northeast Normal University, Changchun, 130117, China; State Key Laboratory of Pathogen and Biosecurity, Key Laboratory of Jilin Province for Zoonosis Prevention and Control, Changchun, 130122, China; School of Information Science and Technology, Northeast Normal University, Changchun, 130117, China; School of Information Science and Technology, Northeast Normal University, Changchun, 130117, China; School of Information Science and Technology, Northeast Normal University, Changchun, 130117, China; Academy of Military Medical Sciences, Beijing, 100850, China; Academy of Military Medical Sciences, Beijing, 100850, China

## Abstract

**Motivation:**

Predicting the thermodynamic stability of proteins upon single-point mutations is a pivotal step in both protein engineering and medicine. In the study of predicting protein thermodynamic stability, various computational methods, whether they extract features at the local-level or global-level, exhibit their respective advantages and limitations. To leverage the advantages of both features, we developed MuFaDDG, a novel sequence-based method that integrated multiscale feature fusion for improved prediction of protein stability changes (ΔΔG).

**Results:**

MuFaDDG achieves comparable performance on the S669 benchmark, demonstrating strong capabilities in stabilizing mutations. Notably, it shows a significant advantage in the ACC metric, with values of 0.75, 0.88, and 0.81 on the direct, reverse, and overall datasets of the CAGI5 Challenge’s Frataxin, respectively. Furthermore, our method outperforms leading sequence-based approaches including THPLM, DDGemb, DDGun, and INPS-Seq on protein Myoglobin stability prediction. Additionally, MuFaDDG demonstrates exceptional predictive performance with higher PCC and ACC on the protein ThreeFoil, which is uncurated by FireProtDB and ProThermDB databases.

**Availability and implementation:**

The source code and data are available at https://github.com/PengjiaMa23/MuFaDDG.

## 1 Introduction

The thermodynamic stability of proteins significantly impacts the function and structure of proteins within organisms, as well as protein engineering, such as industrial biocatalysts (Chapman et al. 2018, Wu et al. 2021, Bell et al. 2021) and pharmaceutical biologics (Gebauer and Skerra 2009, Jay and Lee 2013, Karmakar et al. 2020, Portelli et al. 2021). Decreased thermodynamic stability may lead to increased protein denaturation, thereby affecting biological functions, including enzymatic activity, signal transduction, and structural integrity. On the other hand, proteins with enhanced thermodynamic stability are more amenable to engineering, as they are less prone to unfolding and aggregation (Benevenuta et al. 2022, Rahban et al. 2022, Diaz et al. 2024). To get the more stable candidate protein, computational methods and mutagenesis techniques are proposed to identify advantageous point mutations (Planas-Iglesias et al. 2021). Among them, computational methods for predicting mutant stability can significantly save costs and time by helping prioritize the most promising mutants for experimental characterization, such as direct evolution (Wang et al. 2021).

Existing computational algorithms for predicting protein thermodynamic stability changes can be roughly divided into two main approaches: structure-based methods and sequence-based methods (Benevenuta et al. 2021, Li et al. 2021, Montanucci et al. 2022). Structure-based methods, such as physics-based methods ABACUS (Xiong et al. 2017), Rosetta (Das and Baker 2008) and FoldX (Schymkowitz et al. 2005), are commonly used in the community. Due to their foundation in physical principles, they tend to offer highly accurate predictions, particularly when high-quality structural data is available. Some studies (Kurniawan and Ishida 2023, Lee et al. 2025, Rossi et al. 2025) have accounted for the free energy changes of the unfolded state, as Rossi et al. (2025) demonstrated that incorporating mass balance as a first approximation of the unfolded state significantly improves some physics-based methods. Recent advancements in machine learning have led to the structure-based predictors’ development. Stability Oracle (Diaz et al. 2024) based on graph-transformer, ThermoMPNN (Dieckhaus et al. 2024) based on pre-trained ProteinMPNN (Dauparas et al. 2022) and PremPS (Zhang et al. 2020) based on random forest (RF) regression are proposed and achieved the state-of-the-art performance on one or more testing datasets from protein structures. Structure-based methods typically provide more accurate predictions (Jeffery et al. 2022). However, sequence-based methods have the advantage of not requiring high-quality structural data and being able to efficiently screen large-scale datasets, therefore, they can be used for pre-screening protein variants in early-stage research.

According to reports, following the conclusion of existing prediction approaches and tools on protein stability changes (Table 1, available as supplementary data at *Bioinformatics* online), sequence-based methods occupy only a small portion of these methods. Sequence-based computational approaches for feature extraction emphasize different aspects and can be broadly categorized into residue-level methods (local-level methods) and protein-level methods (global-level methods). Residue-level methods mostly focus on features related to enthalpy changes, primarily relying on local-level features that represent the attractive forces between residues, such as changes in secondary structure and variations in the physicochemical properties of amino acids. The representation works include MU3DSP (Gong et al. 2023), MUpro (Cheng et al. 2006), SAAFEC-SEQ (Li et al. 2021), INPS-Seq (Fariselli et al. 2015), and DDGun (Montanucci et al. 2019). Protein-level methods DDGemb (Savojardo et al. 2024) and THPLM (Gong et al. 2023) represents the protein thermodynamic stability change in view of the global sequence by protein language model, which incorporates knowledge about protein flexibility and disorder (Kabir and Hoque 2024) through its learned representation of protein sequence. Designing a protein with the desired stability is a challenging task. The stability changes of proteins upon single-point mutations are influenced by various factors at different scales. Therefore, extracting and integrating features from both local and global perspectives is crucial for comprehensively assessing the stability changes. Local-level features can provide detailed information about the specific mutation site and its surrounding environment, while global-level features can capture the overall characteristics and context of the protein sequence. By combining these two types of features, we can obtain a more comprehensive and accurate representation of the protein’s stability changes.

**Table 1:** Performance comparison of stability prediction methods on protein ThreeFoil.

Method	RMSE (kcal/mol)	PCC	ACC
MuFaDDG	**1.81**	**0.64**	**0.60**
DDGemb	1.00	0.15	0.40
INPS-Seq	1.22	0.01	0.60
DDGun	1.48	−0.12	0.60
THPLM	1.07	−0.35	0.10

Here, we developed a thermodynamic stability prediction tool named MuFaDDG, which aimed to be used for pre-screening proteins or protein variants from sequences in early-stage research in protein engineering. MuFaDDG is a multiscale feature fusion method that integrates both global-level changes and local residue alterations before and after protein single-point mutations. It combines ESM-2's global sequence representations with NetSurfP-3.0 (Høie et al. 2022) predicted local structural features around mutation sites. The fused features’ model MuFaDDG outperformed models based on single-scale features and demonstrated comparable and even superior performance compared to other sequence-based methods when tested on commonly used external testing set S669, the CAGI5 Challenge’s Frataxin, the protein Myoglobin and the protein ThreeFoil which not curated by both FireProtDB and ProThermDB databases. Moreover, MuFaDDG demonstrated a better performance in stabilizing mutations that are naturally less common.

## 2 Methods

### 2.1 Dataset curation and preprocessing

For a fair performance comparison against the methods for predicting ΔΔG, we used widely recognized public benchmarking datasets as follows. S2648 (Dehouck et al. 2011, Fariselli et al. 2015) served as the training set for training the MuFaDDG model. Considering the antisymmetric characteristic of the Gibbs free energy difference before and after mutation, S2648 encompasses both forward and reverse mutations. For effective cross-validation, Fariselli et al.'s method (Fariselli et al. 2015) was employed, segmenting S2648 based on sequence homology into five parts for training.

S669 (Pancotti et al. 2022) and S^sym^ (Pucci et al. 2018) were designated as testing sets. S669, sourced from the latest ProTherm database (Nikam et al. 2021), contains variations with less than 25% homology to the sequences in S2648. $S^{\mathrm{sym}}$ comprises PDB structures for forward and reverse mutants and has been previously employed to evaluate anti-symmetry and bias across different methods (Mishra 2022).

### 2.2 MuFaDDG architecture

**Figure 1:**
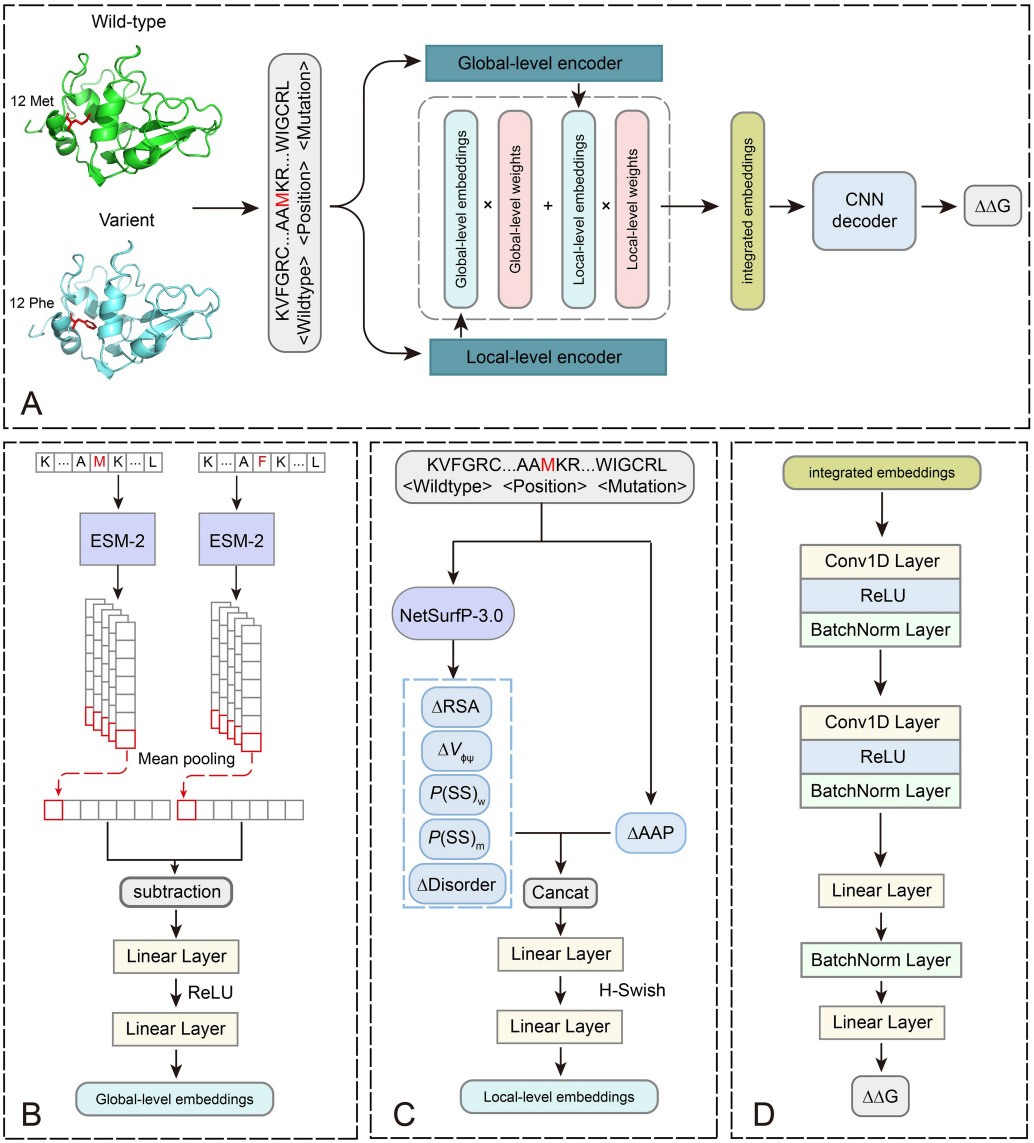
Architecture of the MuFaDDG model. (A) Overview of the MuFaDDG model, comprising two feature extractors (global-level and local-level encoders), an integrated module, and a decoder. (B) Global-level encoder. (C) Local-level encoder. (D) Decoder: Composed of convolutional and fully connected layers, the decoder predicts the ΔΔG value of the mutation.

In this study, we developed a novel model, named MuFaDDG, which combined two feature extractors, an integrated module and a decoder, aimed at accurately predicting ΔΔG upon single-point mutations (Fig. 1A). The overall architecture of MuFaDDG is illustrated in Fig. 1.

Two feature extractors, including local-level and global-level encoders, are designed to characterize changes associated with mutations. On a global scale (sequence-level), embeddings of amino acid sequences before and after mutation are acquired separately through the pre-trained protein language model ESM-2. These embeddings are then subjected to average pooling across the amino acid residue dimension, and the difference between the pre- and post-mutation embeddings is calculated to obtain a global-level feature representation (Fig. 1B). To represent local-level changes, we computed amino acid properties changes (ΔAAP) of the mutant residue and used NetSurfP (Høie et al. 2022) to obtain a set of structural and physicochemical features, including secondary structure probabilities (P(SS)) for both wild-type and mutant residues, changes in relative solvent accessibility (ΔRSA), disorder propensity (ΔDisorder), and dihedral angle conformations (ΔV_ΦΨ_). These features are combined to form preliminary local-level features, which are then refined through two linear layers to achieve a representation of local-level characteristics. Next, the integrated module used the linear combination to leverage the local-level and global-level representations (Fig. 1, available as supplementary data at *Bioinformatics* online). Then, the decoder comprises two convolutional layers and two fully connected layers with three BatchNorm layers for predicting ΔΔG.

### 2.3 Feature representation and fusion

**Global-level encoder.** Protein language models (PLMs) have seen significant advancements in recent years (Rives et al. 2021, Elnaggar et al. 2022, Madani et al. 2023, Su et al. 2024). Specifically, ESM-2 is a transformer-based model that has shown remarkable performance in capturing the evolutionary information of protein sequences, which is crucial for understanding their functions.

In this study, the global-level encoder consists of an ESM-2 backbone and two linear layers, which generates 128-dimensional embeddings. Considering hardware constraints and model capabilities, a pre-trained ESM-2 model with 650 million parameters (esm2_t36_650M_UR50D) was selected. Protein sequences before and after mutation were input into the ESM-2 model, obtaining high-dimensional embeddings with a dimensionality of L×1280 for both the wild-type (${\text{Seq}}_{w}$) and mutant (${\text{Seq}}_{m}$) proteins. L denotes the sequence length.

In this study, the global-level encoder consists of an ESM-2 backbone and two linear layers, which generates 128-dimensional embeddings. Considering hardware constraints and model capabilities, a pre-trained ESM-2 model with 650 million parameters (esm2_t36_650M_UR50D) was selected. Protein sequences before and after mutation were input into the ESM-2 model, obtaining high-dimensional embeddings with a dimensionality of L×1280 for both the wild-type (${\text{Seq}}_{w}$) and mutant (${\text{Seq}}_{m}$) proteins. L denotes the sequence length.

We applied average pooling to reduce the impact of individual amino acid positions while emphasizing global changes, resulting in a unified representation with a dimension of 1×1280. The global-level feature representation $G_{\mathrm{pri}}$ was then obtained by calculating the difference between the wild-type and mutant embeddings. The formulation is as follows:


$$G_{\mathrm{pri}}=\mathrm{AvgPool}(\mathrm{ESM}({\mathrm{Seq}}_{m}))-\mathrm{AvgPool}(\mathrm{ESM}({\mathrm{Seq}}_{w}))$$

$$G=w_{2}(\sigma (w_{1}G_{\mathrm{pri}}+b_{1}))+b_{2}$$

Next, to effectively integrate and analyze both local-level and global-level representations, enabling the model to capture and utilize the correlations and complementarities between various representations, we adjusted the dimensionality of the global change features to 1×128 through two linear layers.

**Local-level encoder.** The local-level encoder is composed of two linear layers that transform 25-dimensional manually extracted features into 128-dimensional local embeddings. The manually extracted features are primarily focused on and centred around mutated residues, including 13-dimensional changes of amino acid properties (ΔAAP), 6-dimensional secondary structure probabilities (P(SS)), 1-dimensional relative solvent accessibility changes (ΔRSA) at the mutation site, 1-dimensional disorder propensity changes (ΔDisorder), and 4-dimensional dihedral angle conformational changes (ΔV_ΦΨ_), all of them are the decisive factors for stability (Folkman et al. 2016, Liu et al. 2019, Gong et al. 2023).

The P(SS), ΔDisorder, ΔRSA and ΔV_ΦΨ_ features are obtained by NetSurfP-3.0, where P(SS) includes probability values for α-helix, β-sheet, and coil states for both wild-type and mutant residues and Δhere P(SS) includes probability values for α-helix, β-sheet, and coil states for both wild-type and mutant residues and CYP119.


$$\mathrm{RSA}=\frac{\mathrm{ASA}}{{\mathrm{ASA}}_{m}}$$

$$\Delta \mathrm{RSA}={\mathrm{RSA}}_{m}-{\mathrm{RSA}}_{w}$$

To address angular periodicity artifacts, the backbone dihedral angle changes ΔV_ΦΨ_ are represented using sine/cosine differences:


$$V_{\Phi \Psi }=\;[\text{sin}(\Phi ),\;\text{cos}(\Phi ),\;\text{sin}(\Psi ),\;\text{cos}(\Psi )]$$

$${\Delta V}_{\Phi \Psi }=V_{\Phi \Psi }^{m}-V_{\Phi \Psi }^{w}$$

These features are concatenated into a 25-dimensional local residue representation, which is then processed through two linear layers:


$$\text{Res}=\;[\Delta \mathrm{RSA},{\Delta V}_{\Phi \Psi },\Delta \mathrm{Disorder},\Delta \mathrm{AAP},{P(SS)}_{w},{P(SS)}_{m}]$$

$$R=w_{2}(\sigma (w_{1}{\text{Res}}_{x}+b_{1}))+b_{2}$$

Res refers to the 25-dimensional manually extracted features. $w_{1}$and $w_{2}$ represent the weight matrices of the two linear layers, while $b_{1}$ and $b_{2}$ are the bias terms. The symbol σ denotes the H-Swish activation function defined by the following expression:


H-swish(x)=x ReLU6(x+3)/6

ReLU6(x)=min(max(0,x),6)

Finally, Res can be mapped into a 128-dimensional local-level feature representation, denoted as *R* (Local-level representations around residue).

### 2.4 Features integration and convolution neural network (CNN) decoder

Following the encoders, we acquired both local-level and global-level representations, each with a dimensionality of 1 × 128. Next, we integrated the local feature change embeddings and global feature change embeddings into a unified representation for prediction. After exploring three methods for combining these embeddings, Concatenation, Outer Product and Linear Combination (detailed in Table 2, Fig. 1, available as supplementary data at *Bioinformatics* online), we ultimately selected the linear combination method for integrating features. The integrated new representation is denoted as $f_{\mathrm{Integrated}}$:


$$f_{\;\mathrm{Integrated}}=\;G\circ G\_\mathrm{weight}+\;R\circ R\_\mathrm{weight}$$

Here, G represents the dimensionally adjusted global-level representation, and R denotes the local-level representation. G_weight and R_weight are two learnable weight matrices, each with dimensions of 1×128. The symbol ∘ represents the Hadamard product, facilitating the element-wise multiplication between the two matrices.

Convolution neural network (CNN) Decoder is a regression model for predicting ΔΔG with two CNN layers and two linear layers (Fig. 1D). The CNN layer is composed of a 1-dimensional convolution operation (Conv1d), followed by a Rectified Linear Unit (ReLU) activation function and a batch normalization (BN). Two CNN layers can be represented as follows:


$$E_{1}=\mathrm{ReLU}(BN(\mathrm{Conv}1d(f_{\mathrm{integrated}})))$$

$$E_{2}=\mathrm{ReLU}(BN(\mathrm{Conv}1d(E_{1})))$$

Then, $E_{2}$ is passed through two linear layers (Linear) to obtain the final predictive result, denoted as $\hat{y}$.


$$\hat{y}=\mathrm{Linear}(BN(\mathrm{Linear}(\mathrm{flatten}(E_{2}))))$$

### 2.5 Evaluation metrics

For the evaluation of our proposed MuFaDDG, we used Root Mean Square Error (RMSE), Accuracy (ACC), Pearson Correlation Coefficient (PCC), antisymmetric property ($r_{d-r}$) and bias property (<δ>) as evaluation metrics. RMSE measures the difference between predicted and experimental ΔΔGs, while PCC assesses the degree of linear correlation between the predicted and experimental ΔΔGs. ACC evaluates the binary classification performance by categorizing mutations as stabilizing (ΔΔG ≥ 0) or destabilizing (ΔΔG < 0). Moreover, $r_{d-r}$ and  < δ> between the predicted ΔΔGs of the direct and corresponding reverse variants were used to evaluate the model’s ability to anti-symmetric mutation (Mishra 2022).

## 3 Results

### 3.1 Cross-validation performance of MuFaDDG

**Figure 2:**
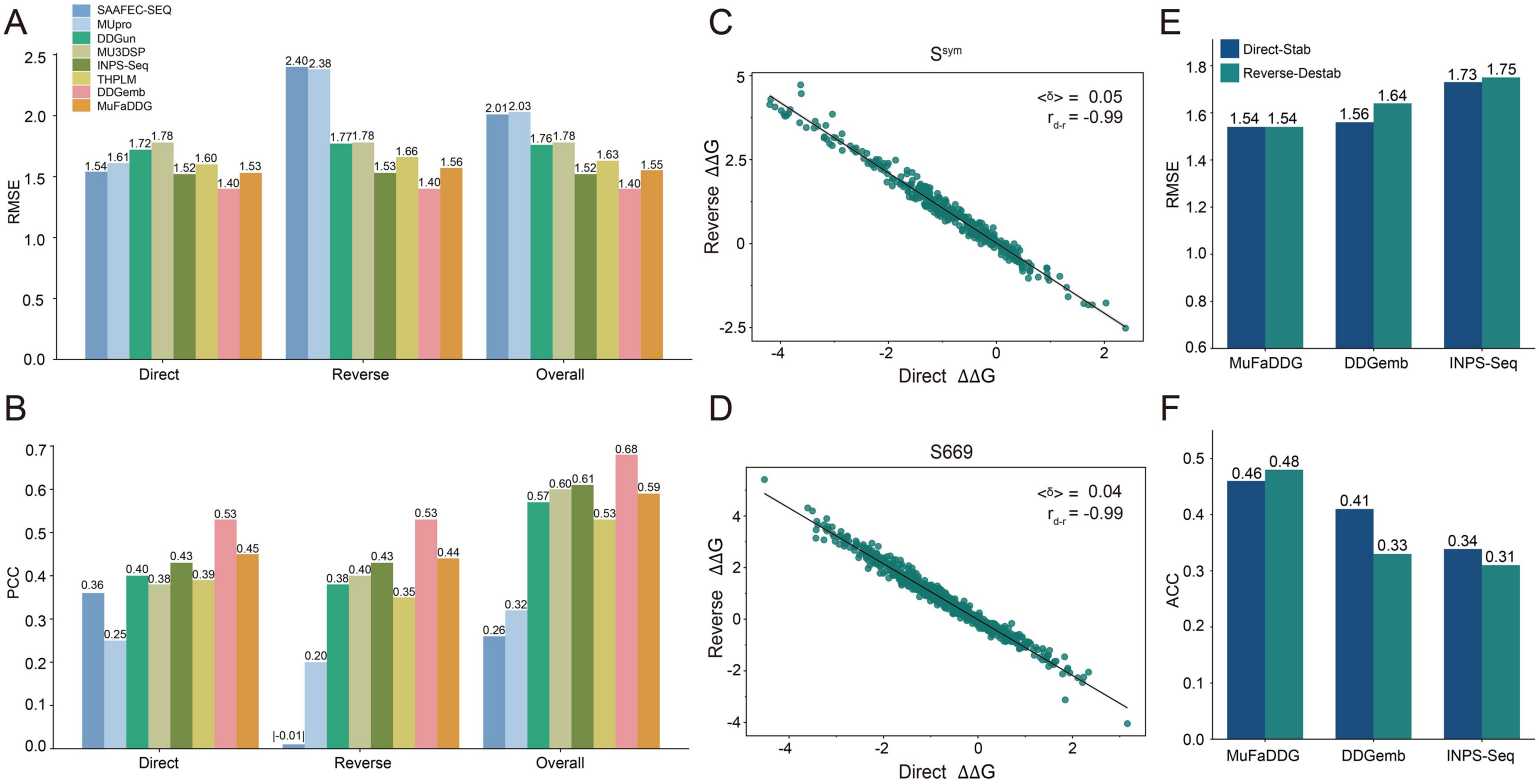
Comparison of MuFaDDG with other sequence-based models on two test sets. (A) RMSE of eight sequence-based methods on the S669 dataset. (B) PCC of eight sequence-based methods on the S669 dataset. (C) Bias and antisymmetry properties of MuFaDDG on the S^sym^ dataset. (D) Bias property and antisymmetry properties of MuFaDDG on the S669 dataset. RMSE (E) and ACC (F) comparison of MuFaDDG, INPS-Seq and DDGemb on the S669 dataset’s forward stabilizing mutations and reverse destabilizing mutations.

To evaluate the performance and generalizability of the MuFaDDG model, cross-validation is employed by testing it on different subsets of data. The S2648 dataset was partitioned into five cross-validation sets based on homology criteria proposed by Fariselli et al.(Dehouck et al. 2011). We trained five independent cross-validation models and integrated them into a composite model by averaging, which we named MuFaDDG. Specifically, the five-fold cross-validation yielded PCC of 0.74, 0.62, 0.76, 0.61, and 0.74 for the individual models, with an average PCC of 0.69 (Fig. 2, available as supplementary data at *Bioinformatics* online). The corresponding RMSE of overall variations were 1.25 kcal/mol, 1.33 kcal/mol, 1.25 kcal/mol, 1.46 kcal/mol, and 1.22 kcal/mol, resulting in an average RMSE of 1.30 kcal/mol (Table 3, available as supplementary data at *Bioinformatics* online).

### 3.2 Performance comparison on datasets S669

To validate the robustness of the MuFaDDG model, we compared it with seven advanced sequence-based predictors, THPLM, MU3DSP, DDGun, INPS-Seq, MUpro, SAAFEC-SEQ, and DDGemb on the S669 testing sets. Considering the antisymmetric property of predictors, S669 is divided into direct, reverse, and overall mutation datasets to evaluate these predictors by RMSE and PCC metrics, which are shown in Fig. 2.

On the S669 dataset (Fig. 2A and B), MuFaDDG showed compara-ble performance with other methods in terms of RMSE and PCC on the S669 direct, reverse, and overall mutation sets. Notably, MuFaDDG achieved the second-best PCC on both direct (PCC = 0.45) and reverse (PCC = 0.44) mutations. It also obtained the top 3 RMSE on direct (MuFaDDG = 1.53 kcal/mol, INPS-Seq = 1.52 kcal/mol, DDGemb = 1.40 kcal/mol), reverse (MuFaDDG = 1.56 kcal/mol, INPS-Seq = 1.53 kcal/mol, DDGemb = 1.40 kcal/mol), and overall mutations (MuFaDDG = 1.55 kcal/mol, INPS-Seq = 1.52 kcal/mol, DDGemb = 1.40 kcal/mol). In addition, MuFaDDG exhibited strong antisymmetry and low bias, with an antisymmetric property of -0.99 and a bias property (<δ>) of 0.04 kcal/mol on dataset S669 (Fig. 2D) as well as an antisymmetric property of -0.99 and a bias property (<δ>) of 0.05 kcal/mol on dataset S^sym^ (Fig. 2C).

Notably, stabilizing mutations are naturally less common (Nisthal et al. 2019) and many methods suffer from poor generalization on stabilizing mutations (Diaz et al. 2024). Therefore, we further evaluated the performance of MuFaDDG, DDGemb and INPS-Seq on direct stabilizing mutations of S669. In Fig. 2E and F, MuFaDDG achieved a better performance than INPS-Seq and DDGemb with an ACC of 0.46 (INPS-Seq = 0.34, DDGemb = 0.41) and a RMSE of 1.54 kcal/mol (INPS-Seq= 1.56 kcal/mol, DDGemb = 1.73 kcal/mol) on direct stability mutations, respectively. A detailed comparison of MuFaDDG with other sequence-based predictors on stabilizing and reverse destabilizing mutations in the S669 dataset is provided in Table 4, available as supplementary data at *Bioinformatics* online.

### 3.3 MuFaDDG prediction preference analysis

To evaluate the applicability spectrum of MuFaDDG, we analyzed its prediction preferences, including the relative solvent accessibility (RSA) and secondary structure of the mutated residues to determine which type of mutated residues that MuFaDDG predicts more effectively. Secondary structure (Ji and Li 2010) refers to the specific structural motifs (like α-helices, β-sheets, and loops) that play key roles in the stability of proteins, while RSA reflects the extent to which residues are exposed to the solvent (typically water) within the protein structure. The secondary structure and RSA of each mutated residue were obtained by the DSSP program (Joosten et al. 2011). In particular, residues with RSA greater than 0.5 are defined as solvent-exposed residues, while those with RSA less than 0.1 are defined as being buried within the protein.

**Figure 3:**
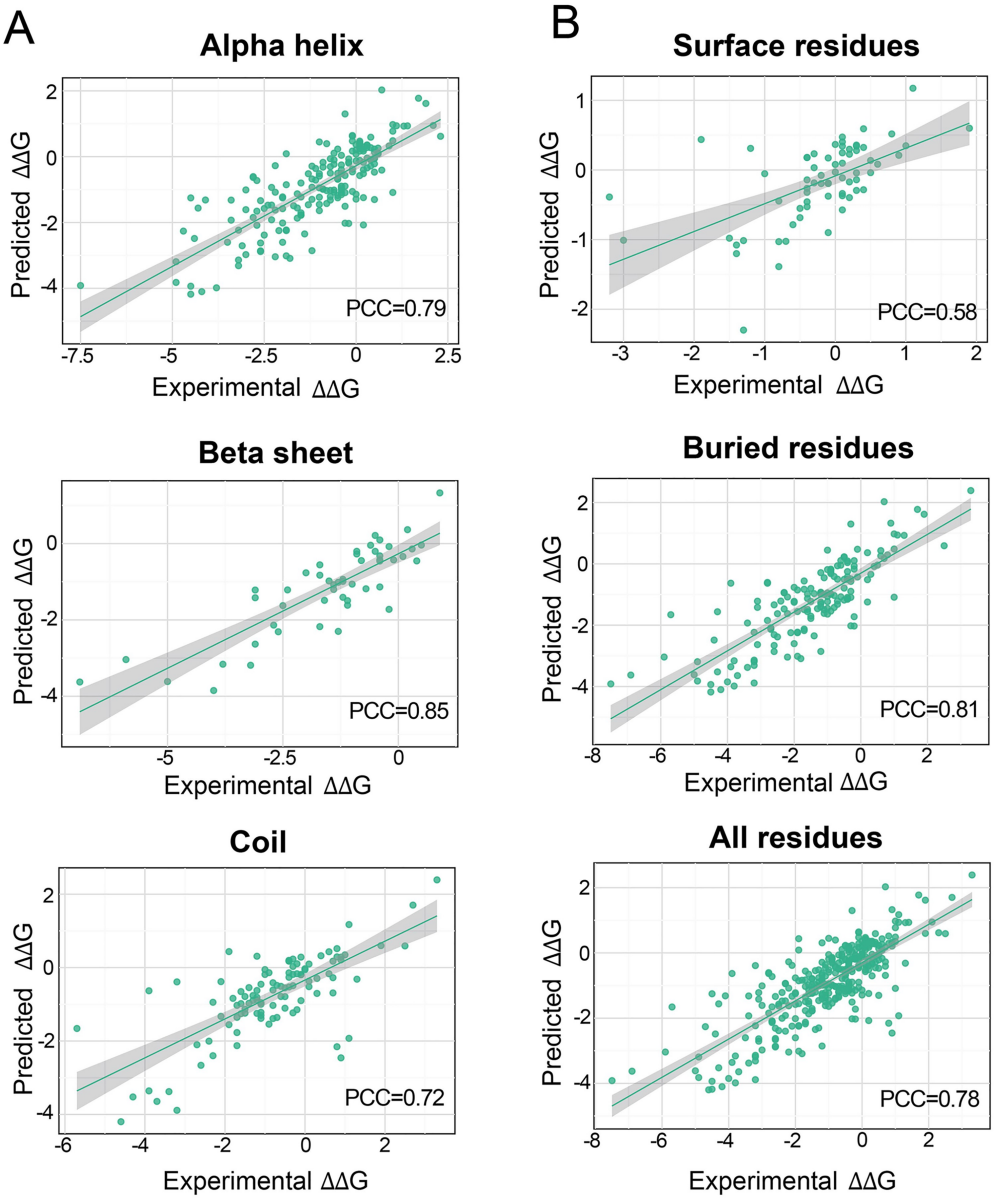
Analysis of secondary structure and mutation residue position. (A) Performance of MuFaDDG on different secondary structures in the S^sym^ dataset. (B) Performance of MuFaDDG on different mutation residue positions in the S^sym^ dataset.

Fig. 3A and Fig. 3B show the predictive performance of MuFaDDG on residues of different secondary structures located in different positions within the proteins in the S^sym^ testing sets. Under different secondary structures, MuFaDDG performs best in predicting mutations in residues with β-sheet structures (PCC = 0.85) and shows slightly poorer performance for α-helix (PCC = 0.79) and random coil (PCC = 0.72) structures (Fig. 3A). In terms of different mutation residue positions, Fig. 3B demonstrates that MuFaDDG predictive performance is better for buried residues (PCC = 0.81) than for surface residues (0.58). A possible reason is that mutations in surface residues might also need to consider environmental factors such as solvents, and the model lacks this type of information in its input features, leading to poorer performance in predicting thermodynamic stability changes caused by mutations in protein surface residues.

### 3.4 Ablation study

**Figure 4:**
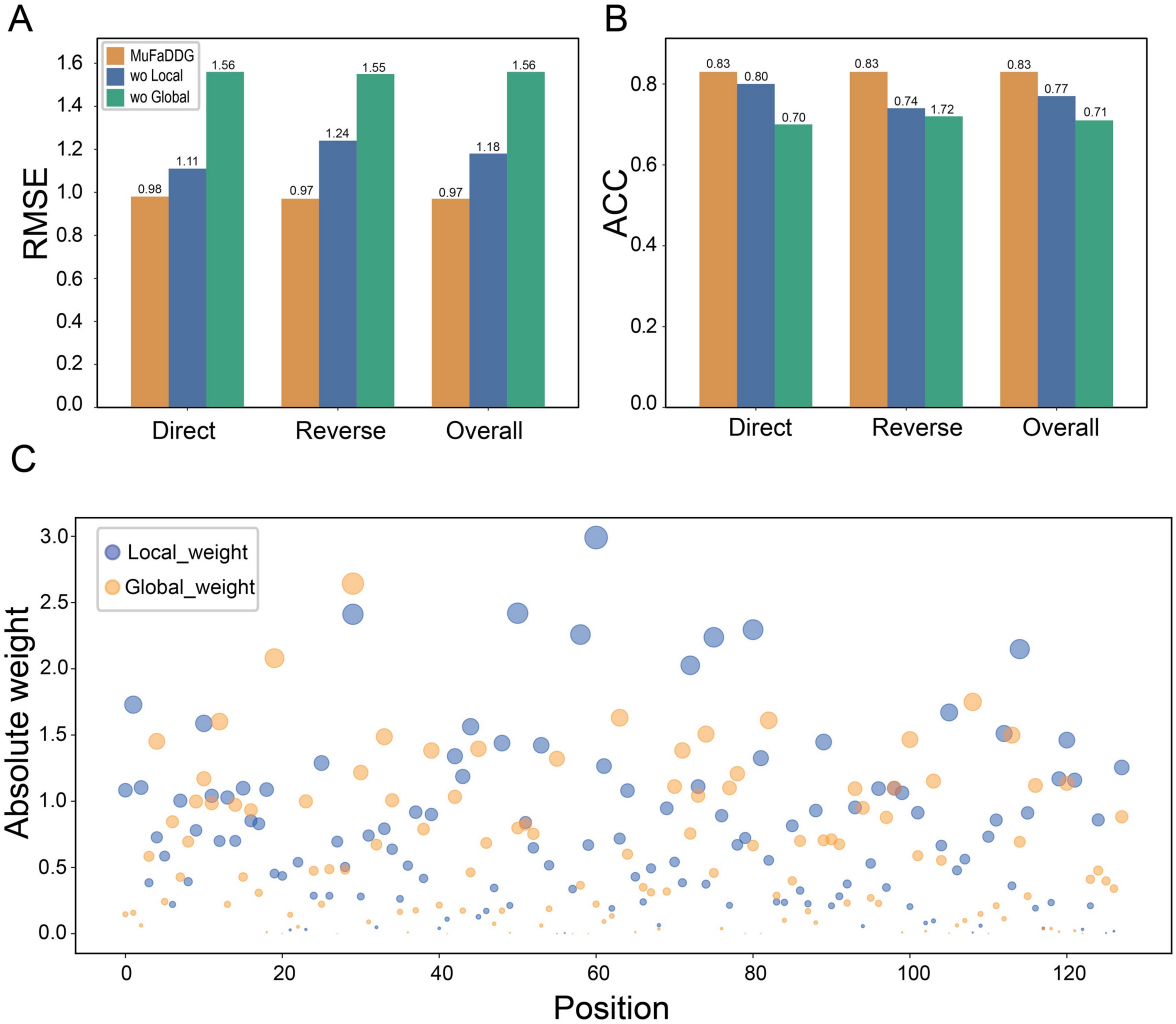
Ablation experiments, and feature matrix visualization. (A) Ablation study results of MuFaDDG on the S^sym^ test set, showing the RMSE metric. (B) Ablation study results of MuFaDDG on the S^sym^ test set, showing the ACC metric. (C) Visualization of global and local feature matrix weights in MuFaDDG.

To determine whether combining global-level features with local-level features contributes to MuFaDDG’s performance, we conducted an ablation study by removing one of them. We found MuFaDDG significantly outperforms the other two models (Fig. 4A and B), which were trained using a single type of feature. Specifically, the ablated model 'wo Global’ (excluding global features) retained only the local residue feature extraction module, whereas 'wo Local’ (excluding local features) kept only the global-level encoder module. Importantly, MuFaDDG demonstrates significant improvements over the ablated models. Compared to 'wo Local’, it achieves 3.75%, 12.16%, and 7.79% higher ACC on the direct, reverse, and overall S^sym^ datasets, respectively. Compared to 'wo Global’, it shows 18.57%, 15.27%, and 16.90% ACC improvements on the corresponding datasets. Detailed comparative data from the ablation studies are provided in Table 5, available as supplementary data at *Bioinformatics* online.

Since 'wo Local’ outperformed 'wo Global’ in terms of PCC and RMSE on the direct, reverse, and overall datasets (Fig. 4A and B), we further analyzed the contribution of these two feature types in the MuFaDDG model. Fig. 4C demonstrates the absolute weights on 128-dimensional embeddings when integrating both feature types, revealing which features ('wo Global’ or 'wo Local’) contribute more significantly to predicting protein stability changes. It shows that the model attends to both local structural features and global sequence features, while assigning slightly greater importance to local structural features. This weighting pattern indicates that the model effectively captures meaningful information from protein structural features while maintaining a balanced consideration of global sequence characteristics.

### 3.5 The effect of mutations on a three-fold symmetric globular protein ThreeFoil

ThreeFoil is a three-fold symmetric globular protein by computational reconstructed and repeated the β-trefoil subdomain module (Broom et al. 2012). ThreeFoil is not recorded in both FireProtDB (Stourac et al. 2021) and ProThermDB (Nikam et al. 2021) databases, and it displays lower 30% identity similarity with training dataset S2648. Therefore, we applied MuFaDDG on protein ThreeFoil that contained ten mutations consisting of three no significant effect mutations, four stabilizing mutations, and three destabilizing mutations (Broom et al. 2017). As Table 1 demonstrated that our model outperformed other predictors including THPLM, INPS-Seq, DDGemb and DDGun reaching PCC of 0.64 and ACC of 0.60, further confirming the model’s generalization ability.

### 3.6 Performance comparison on CAGI5 challenge’s Frataxin

We further evaluated MuFaDDG using the Frataxin dataset from the CAGI5 challenge (Fowler and Fields 2014). MuFaDDG was compared with four leading sequence-based predictors (THPLM, DDGemb, INPS-Seq, and DDGun) that considered the ΔΔG prediction of direct and reverse variations across the direct, reverse, and overall datasets.

**Figure 5:**
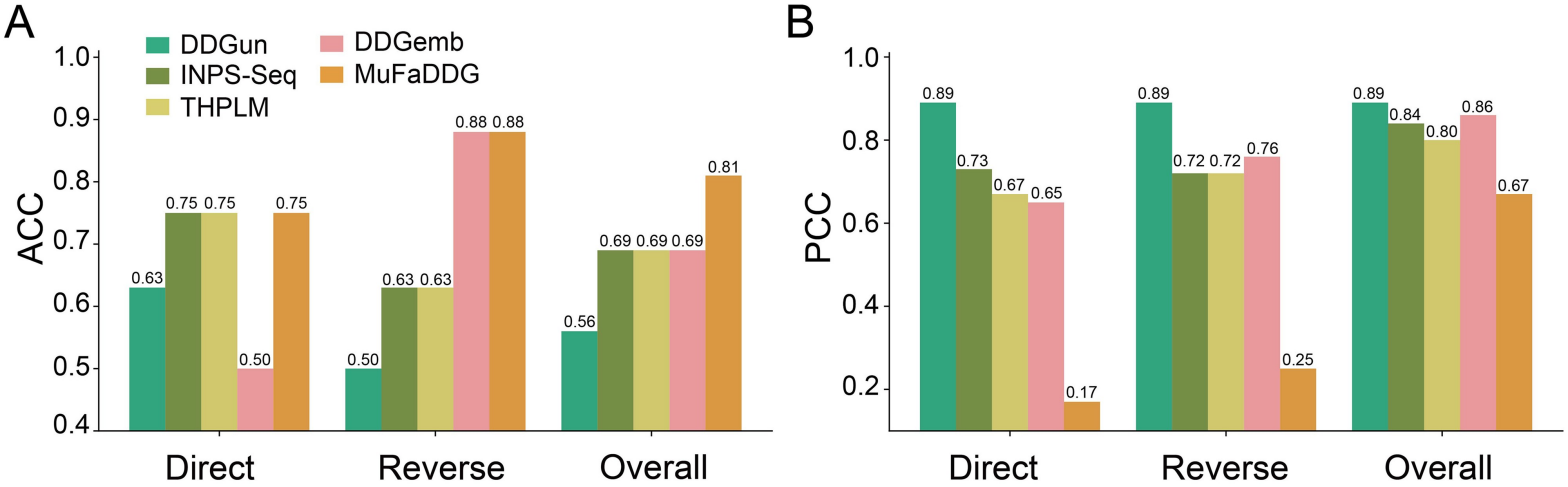
Performance comparison of sequence-based stability changes prediction methods on CAGI5 Challenge’s Frataxin.

The results (Table 6, available as supplementary data at *Bioinformatics* online, Fig. 5) demonstrate that although MuFaDDG slightly underperforms in the PCC metric compared to some methods, it exhibits a significant advantage in the ACC by achieving values of 0.75, 0.88, and 0.81 on the direct, reverse, and overall datasets, respectively.

### 3.7 Achieves the best performance on stability prediction of myoglobin

**Figure 6:**
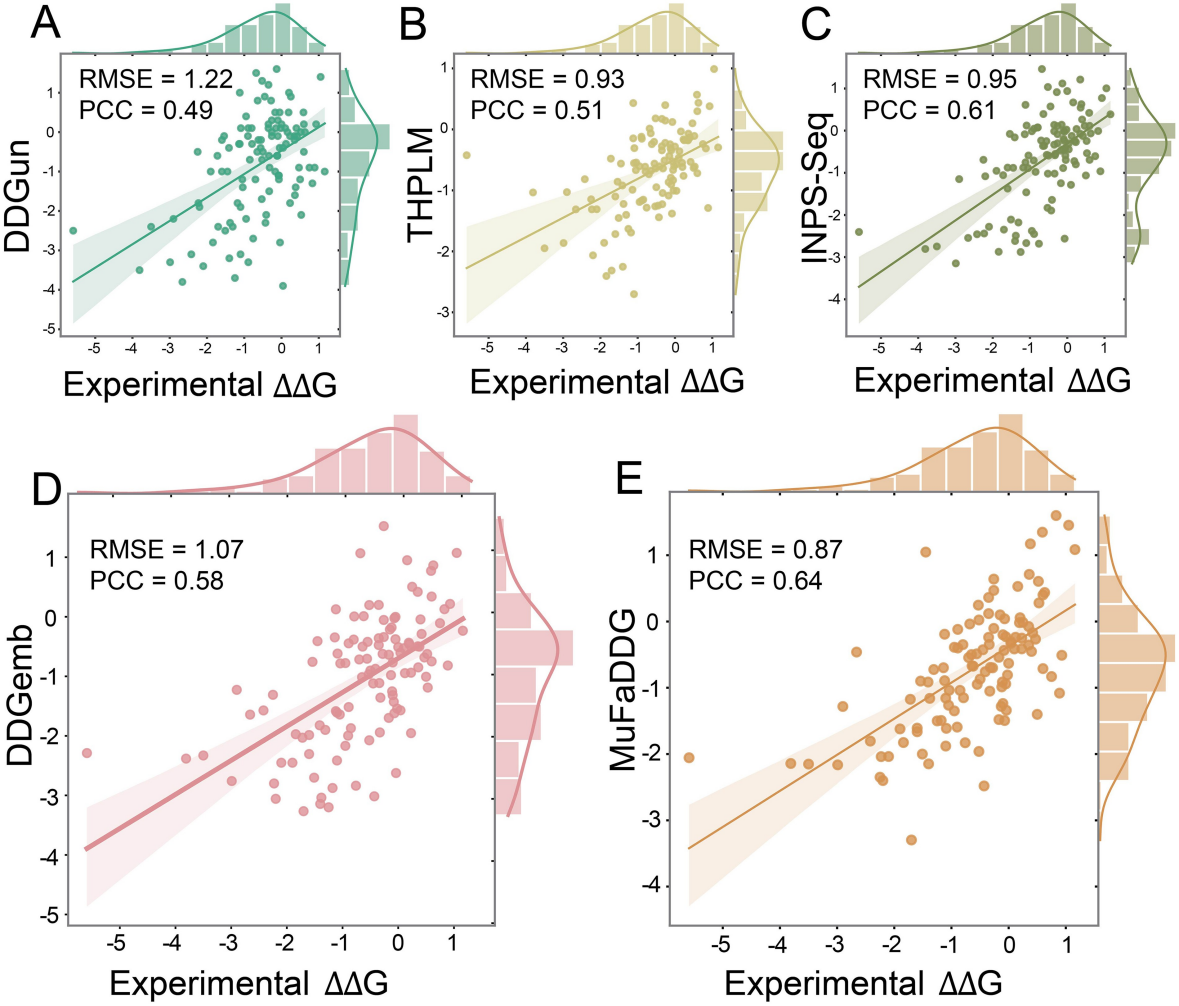
MuFaDDG and other sequence-based methods including THPLM, DDGun, DDGemb and INPS-Seq prediction performance on stability changes of Myoglobin.

Myoglobin, a crucial oxygen-binding protein in muscle tissues, serves as an excellent model for studying protein stability. We evaluated MuFaDDG on a myoglobin dataset derived from Umerenkov et al.'s work (Umerenkov et al. 2023). The ΔΔG values of duplicate mutations from myoglobin dataset were averaged, adhering to the methodology of the training dataset (Dehouck et al. 2011), to ensure data uniformity and yield a final set of 113 unique variants. As shown in Fig. 6, MuFaDDG outperformed all sequence-based methods including THPLM, DDGun, DDGemb and INPS-Seq across all metrics, achieved an RMSE of 0.87 Kcal/mol, PCC of 0.64, and ACC of 0.76, respectively. These results demonstrated MuFaDDG’s robust capability in predicting Myoglobin stability changes, confirming its utility for practical applications in protein engineering.

## 4 Conclusion

In this work, we present MuFaDDG, a novel sequence-based method trained by integrating manually extracted features around mutant residues (local-level features) and ESM2’s representations of variants (global-level features). Our model reports second-best performance with other methods in direct, reverse and overall variations of S669 datasets and superior performance in direct stabilizing variations of S669 (Fig. 2A and B). For further analysis, mutations located in β-sheets and buried residues demonstrated a higher performance, which is easier captured by the feature extraction methods of MuFaDDG. In addition, to validate MuFaDDG, we applied it to three experimentally validated proteins (case studies) that were excluded from the training dataset. The first case study focuses on the CAGI5 Challenge’s Frataxin. MuFaDDG demonstrated a significant advantage to classify stabilizing and destabilizing mutations with 0.81 of ACC on overall variations. The second protein is the Myoglobin. Our model outperformed leading sequence-based methods including THPLM, DDGun, DDGemb, and INPS-Seq across PCC and RMSE on Myoglobin with an improvement from 0.61(INPS-Seq) to 0.64. The rest is a three-fold symmetric globular protein ThreeFoil which is uncurated in FireProtDB and ProThermDB databases. Our model outperformed other predictors including THPLM, INPS-Seq, DDGemb, and DDGun reaching ACC of 0.64 and PCC of 0.60. All in all, MuFaDDG fusing both local and global features demonstrated a comparable robustness and generalization ability for predicting thermodynamic stability changes on low-homology sequences.

## Supplementary Material

btag196_Supplementary_Data

## Data Availability

The source code and data are available at https://github.com/PengjiaMa23/MuFaDDG.
