# Supplementary material for: MuFaDDG: a sequence-based multiscale feature fusion framework for protein stability changes prediction
Source: Bioinformatics. 2026 Apr 29;42(5):btag196. doi: 10.1093/bioinformatics/btag196 (PMC13186199; doi:10.1093/bioinformatics/btag196)
Supplement: btag196_Supplementary_Data [file btag196_supplementary_data.docx]

Supplementary materials

**Combination Methods**

We explored three methods for combining these embeddings:

1. Concatenation: Directly concatenating the local feature change embeddings and global feature change embeddings along the second dimension to form a 1x256 dimensional feature vector representation.

2. Outer Product: Calculating the outer product of the local feature embeddings and global feature embeddings, resulting in a 128x128 dimensional feature vector representation.

3. Linear Combination: Setting up two learnable weight matrices, each of size 1x128, and performing an element-wise multiplication of the local feature embeddings and global feature embeddings with their respective weight matrices, then adding the two to obtain a 1x128 dimensional feature vector representation.

Illustrations of the three combination methods are shown in Fig 2, and their performance on the test sets is detailed in Tables 2. After evaluating the performance of the three methods on the test set, we ultimately selected the linear combination method for integrating features. The integrated new feature is denoted as $f_{\mathrm{Integrated}}$:

$$f_{\mathrm{Integrated}}= G\circ G\_weight+ R\circ R\_weight$$

Here, G represents the dimensionally adjusted global feature representation, and R denotes the local residue feature representation. G_weight and R_weight are two learnable weight matrices, each with dimensions of 1x128. The symbol ∘ represents the Hadamard product, facilitating the element-wise multiplication between the two matrices.

**Evaluation Metrics**

For the evaluation of our proposed MuFaDDG, we used RMSE, ACC, PCC, antisymmetric property ($r_{d-r}$) and bias property (<δ>) as evaluation metrics. RMSE measures the difference between predicted and experimental ΔΔGs, while PCC assesses the degree of linear correlation between the predicted and experimental ΔΔGs. ACC evaluates the binary classification performance by categorizing mutations as stabilizing (ΔΔG ≥ 0) or destabilizing (ΔΔG < 0). The formulations of these metrics are as follows:

$$RMSE=\sqrt{\frac{1}{N}\sum_{i=1}^{N} {(y_{i}-\hat{y}_{i})}^{2}}$$

$$PCC=\frac{\sum_{i=1}^{N} (y_{i}-\bar{y})(\hat{y}_{i}-\bar{\hat{y}})}{\sqrt{\sum_{i=1}^{N} {(y_{i}-\bar{y})}^{2}\sum_{i=1}^{N} {(\hat{y}_{i}-\bar{\hat{y}})}^{2}}}$$

$$ACC=\frac{TP+TN}{N}$$

Where N is the number of samples, $y_{i}$ is the experimental ΔΔG, $\hat{y}_{i}$ is the predicted ΔΔG, $i$ represents the $i$ sample in the data set, $\bar{y}$ represents the average of the experimental ΔΔG, and $\bar{\hat{y}}$ represents the average of the predicted ΔΔG. TP/TN denote correct predictions of stabilizing/destabilizing mutations, respectively.

Moreover, $r_{d-r}$ and <δ> between the predicted ΔΔGs of the direct and corresponding reverse variants were used to evaluate the model’s ability to anti-symmetric mutation [1]

Supplementary Tables

***Table 1***. List of structure-based methods to predict thermodynamic stability changes of proteins upon mutations and freely available via web server or software.

| Methods (Year) | Algorithms | Features | Datasets | URLs | 1D/3D | | Run Time (min) |
| --- | --- | --- | --- | --- | --- | --- | --- |
| SDM^[2]^ (2011) | Linear combi- nation | ESSTs, RSA | S1615 | [Access by http://biosig.unimelb.edu.au/duet/stability](http://biosig.unimelb.edu.au/dynamut/) | | 3D | ∼ 2 |
| PoPMuSiC 2.1^[3]^ (2011) | Neural network | RSA, statistical potentials | S2648 | Unavailable | | 3D | - |
| DUET^[4]^ (2014) | Integrated model (SVM) | Consolidated mCSM and SDM | - | <http://biosig.unimelb.edu.au/duet/stability> | | 3D | ∼ 2 |
| mCSM^[5]^ (2014) | Gaussian and random forest | graph‐based signatures and physico chemical patterns | S2648; S1925 | <http://biosig.unimelb.edu.au/mcsm/> | | 3D | ∼ 2 |
| AUTO-MUTE 2.0^[6]^ (2014) | SVM and random forest | Four-Body Statistical Potential | S1417 | <http://binf.gmu.edu/automute/> | | 3D | ∼ 1 |
| INPS3D^[7]^ (2016) | SVM | Properties such as Sub-score, Hyd, and EvolInfo | S2648 | [http://inps.biocomp.unibo.it](http://inps.biocomp.unibo.it/) | | 3D | ∼4 |
| MAESTRO^[8]^  (2016) | ANN agents and SVM | SS, ASA, statistical potentials, and Hyd | S2648; S1925; S1762 | <https://biwww.che.sbg.ac.at/maestro/web> | | 3D | < 1 |
| SDM2^[2]^ (2017) | Linear combination | Updated ESSTs and new residue-conformation and interaction parameters based on SDM | S2690 | <http://marid.bioc.cam.ac.uk/sdm2/prediction> | | 3D | Based on queued jobs |
| PoPMuSiCsym  ^[9]^ (2018) | Neural network | RSA, statistical potentials | S2648 | <https://soft.dezyme.com/query/create/pop> | | 3D | < 1 |
| DynaMut^[10]^  (2018) | Integrated model | Normal mode analysis and graph‐based signatures approach | S2648 | <http://biosig.unimelb.edu.au/dynamut/>. | | 3D | Unstable |
| DDGun3D^[11]^  (2019) | Linear parametric model | BL62, statistical potentials, Hyd, ASA, and EvolInfo | S2648; VariBench | <https://github.com/biofold/ddgun> | | 3D | ∼30 |
| iDeepDDG^[12]^ (2019) | Integrated model | Predicted ΔΔG values from mCSM, SDM, and DUET as additional inputs | S5444 | <http://protein.org.cn/ddg.html> | | 3D | Unknown |
| DeepDDG^[12]^ (2019) | Neural network | ASA, SS, H-bond, and EvolInfo | S5444 | <http://protein.org.cn/ddg.html> | | 3D | Unknown |
| DynaMut2^[13]^ (2020) | Random forest | Structural environment properties, graph‐based signatures approach | S4022 | <http://biosig.unimelb.edu.au/dynamut2/> | | 3D | Unstable |
| ThermoNet^[14]^ (2020) | 3D CNN | Aromatic, positive, negative, hydrophobicity, H-bond donor/acceptor | Q3214 | <https://github.com/gersteinlab/ThermoNet> | | 3D | ∼ 100 |
| PremPS^[15]^ (2020) | Random forest | EvolInfo, RSA, ΔHyd, Hyd, Aromatic, charged, Leu | S2648 | <https://lilab.jysw.suda.edu.cn/research/PremPS/> | | 3D | ∼ 3 |
| BayeStab^[16]^  (2022) | GNN | Atom type, Molecular information. | S2648, Q3214 | https://github.com/HongzhouTang/BayeStab | | 3D | - |
| ProS-GNN^[17]^  (2023) | GNN | Combining biochemistry principles | S2648 | https://github.com/HongzhouTang/Pros-GNN | | 3D | - |
| ThermoMPNN^[18]^（2024） | Pretrained ProteinMPNN | Backbone atoms; 48 nearest neighboring residues | Megascale | https://github.com/Kuhlman-Lab/ThermoMPNN | | 3D | - |
| Stability Oracle^[19]^ (2024) | Graph Transformer | Voxelized molecular representation | CDNA117K,CDNA24k,C2878,T2837 | https://github.com/danny305/StabilityOracle | | 3D | - |
| MUpro^[20]^ (2006) | SVM | SeqInfo, Neighbors Info | S1615 | https://www.ics.uci.edu/~baldig/mutation.html | | 1D | < 1 |
| iPTREE-STAB^[21]^ (2007) | Adaptive boosting | Neighbors Info | S1859 | Access by http://ncblab.nchu.edu.tw/iStable2/ | | 1D | ∼30 |
| EASE-MM^[22]^ (2016) | Integrated models based SVM | 13 properties such as ΔVol, ΔHyd. EvolInfo and predicted SS and RSA | S1676; S236 | https://www.sparks-lab.org/server/ease-mm/ | | 1D | Based on  queued jobs |
| INPS^[23]^  (2016) | SVM | Properties such as Sub-score, Hyd, and EvolInfo | S2648 | http://inps.biocomp.unibo.it | | 1D | ∼4 |
| PON-tstab^[24]^ (2018) | Gradient boosting | Bulkiness, EvolInfo, Similarity features, variation type features, and Neighbors Info | S1564 | http://structure.bmc.lu.se/PON-Tstab/ | | 1D | ∼30 |
| DDGun^[11]^ (2019) | Linear  parametric  model | BL62, statistical potentials, Hyd, RSA, and EvolInfo | S2648; Vari-Bench | https://github.com/biofold/ddgun | | 1D | ∼3 |
| SAAFEC-SEQ^[25]^ (2021) | Gradient boosting | ASA, properties such as H-bond, Δhyd, Vol, Neighbors Info and EvolInfo | S2648 | <http://compbio.clemson.edu/SAAFEC-SEQ/index.php> | | 1D | ∼ 30 |
| THPLM^[26]^  (2023) | CNN | Protein Language model | S2648 | https://github.com/FPPGroup/THPLM | | 1D | ∼ 30 |
| DDGemb^[27]^ (2025) | Transformer | Protein Language model, transformer | S2450 | https://ddgemb.biocomp.unibo.it. | | 1D | - |
| I-Mutant 2.0 ^[28]^ (2005) | SVM | RSA, RI and 'spatial environment' | S1948(3D);  S2087 | <https://folding.biofold.org/i-mutant/i-mutant2.0.html> | | 1D&3D | ∼ 400 |
| iStable ^[29]^ (2013) | Integrated model | SeqInfo, predicted results from other methods | M1311;  M1820; S630 | <http://predictor.nchu.edu.tw/iStable/> | | 1D&3D | ∼3 |
| ELASPIC ^[30]^ (2016) | Ensemble DT | SemiEnergy, EvolInfo and Neighbors Str Info | S3463 | <http://elaspic.kimlab.org/> | | 1D&3D | <1 or No result |
| STRUM ^[31]^ (2016) | SVM | Energy functions, predicted structure, ΔHyd, ΔVol, ΔIP, ΔMW, EvolInfo | Q3421 | <https://zhanggroup.org/STRUM/> | | 1D&3D | - |
| ACDC-NN ^[32]^ | CNN | Pre-train model IvankovDDGun,EvolInfo | S2648, Ssym | <https://github.com/compbiomed-unito/acdc-nn> | | 1D&3D | - |
| iStable2.0 ^[33]^ (2020) | Integrated model | EvolInfo; predictions from I-Mutant, AUTOMUTE, MUPRO, PoPMuSiC, and CUPSAT | S3568 | <http://ncblab.nchu.edu.tw/iStable2/> | | 1D&3D | ∼30 |

**Table 2.** Performance of different feature combinations on the S^sym^ and S669 test sets.

| Combination Methods | Test Set | Direct | | | Reverse | | | Overall | | |
| --- | --- | --- | --- | --- | --- | --- | --- | --- | --- | --- |
|  |  | RMSE | PCC | ACC | RMSE | PCC | ACC | RMSE | PCC | ACC |
| Concatenation | S^sym^ | 0.98 | 0.78 | 0.83 | 0.97 | 0.78 | 0.83 | 0.98 | 0.85 | 0.83 |
|  | S669 | 1.54 | 0.44 | 0.72 | 1.55 | 0.44 | 0.71 | 1.54 | 0.59 | 0.71 |
| Outer Product | S^sym^ | 0.99 | 0.78 | 0.83 | 0.98 | 0.78 | 0.82 | 0.99 | 0.85 | 0.82 |
|  | S669 | 1.53 | 0.45 | 0.73 | 1.53 | 0.45 | 0.72 | 1.53 | 0.59 | 0.72 |
| Linear Combination | S^sym^ | 0.98 | 0.78 | 0.83 | 0.97 | 0.78 | 0.83 | 0.97 | 0.85 | 0.83 |
|  | S669 | 1.53 | 0.45 | 0.73 | 1.56 | 0.44 | 0.72 | 1.55 | 0.59 | 0.72 |

***Table 3.***  Performance of five cross-validation models on the S2648 Training set*.*

| Model | Direct | | | Reverse | | | Overall | | |
| --- | --- | --- | --- | --- | --- | --- | --- | --- | --- |
|  | RMSE | PCC | ACC | RMSE | PCC | ACC | RMSE | PCC | ACC |
| Fold1 | 1.25 | 0.62 | 0.74 | 1.25 | 0.64 | 0.74 | 1.25 | 0.74 | 0.74 |
| Fold2 | 1.30 | 0.52 | 0.73 | 1.36 | 0.51 | 0.73 | 1.33 | 0.62 | 0.73 |
| Fold3 | 1.27 | 0.68 | 0.76 | 1.22 | 0.67 | 0.79 | 1.25 | 0.76 | 0.77 |
| Fold4 | 1.44 | 0.42 | 0.76 | 1.49 | 0.46 | 0.74 | 1.46 | 0.61 | 0.75 |
| Fold5 | 1.22 | 0.74 | 0.76 | 1.25 | 0.56 | 0.74 | 1.22 | 0.74 | 0.76 |
| Mean | 1.29 | 0.56 | 0.75 | 1.32 | 0.55 | 0.75 | 1.30 | 0.69 | 0.75 |

***Table 4.*** Performance comparison of different methods on stabilizing and destabilizing mutations in the S669 dataset

| Mutation type | Evaluation Metrics | Methods | | | | | | |  |
| --- | --- | --- | --- | --- | --- | --- | --- | --- | --- |
|  |  | MuFaDDG | INPS-Seq | SAAFEC-SEQ | MUpro | DDGun | MU3DSP | THPLM | GGDemb |
| Direct-stable | RMSE | 1.54 | 1.73 | 1.99 | 2.20 | 1.95 | 1.46 | 1.64 | 1.56 |
|  | PCC | 0.17 | 0.15 | 0.02 | -0.03 | 0.12 | 0.10 | 0.16 | 0.19 |
|  | ACC | 0.46 | 0.34 | 0.11 | 0.04 | 0.39 | 0.17 | 0.46 | 0.41 |
| Direct-destab | RMSE | 1.53 | 1.44 | 1.35 | 1.35 | 1.67 | 1.88 | 1.58 | 1.34 |
|  | PCC | 0.33 | 0.33 | 0.37 | 0.24 | 0.31 | 0.30 | 0.29 | 0.40 |
|  | ACC | 0.82 | 0.88 | 0.94 | 0.97 | 0.80 | 0.95 | 0.83 | 0.87 |
| Rever-stable | RMSE | 1.57 | 1.44 | 2.69 | 2.66 | 1.71 | 1.87 | 1.62 | 1.31 |
|  | PCC | 0.33 | 0.34 | -0.09 | 0.12 | 0.29 | 0.30 | 0.29 | 0.40 |
|  | ACC | 0.80 | 0.87 | 0.03 | 0.06 | 0.79 | 0.93 | 0.80 | 0.88 |
| Rever-destable | RMSE | 1.54 | 1.75 | 1.15 | 1.21 | 1.96 | 1.46 | 1.78 | 1.64 |
|  | PCC | 0.20 | 0.14 | 0.05 | -0.05 | 0.10 | 0.08 | 0.11 | 0.18 |
|  | ACC | 0.48 | 0.31 | 0.95 | 0.98 | 0.43 | 0.22 | 0.51 | 0.33 |

**Table 5.** Comparative Results of Ablation Studies on S669 and S^sym^ Datasets

| Combination Methods | Test Set | Direct | | | Reverse | | | Overall | | |
| --- | --- | --- | --- | --- | --- | --- | --- | --- | --- | --- |
|  |  | RMSE | PCC | ACC | RMSE | PCC | ACC | RMSE | PCC | ACC |
| wo Local | S^sym^ | 1.11 | 0.77 | 0.80 | 1.24 | 0.76 | 0.74 | 1.18 | 0.84 | 0.77 |
|  | S669 | 1.59 | 0.41 | 0.75 | 1.77 | 0.44 | 0.63 | 1.68 | 0.57 | 0.69 |
| wo Global | S^sym^ | 1.56 | 0.48 | 0.70 | 1.55 | 0.48 | 0.72 | 1.56 | 0.56 | 0.71 |
|  | S669 | 1.61 | 0.40 | 0.69 | 1.59 | 0.41 | 0.69 | 1.60 | 0.54 | 0.69 |
| MuFaDDG | S^sym^ | 0.98 | 0.78 | 0.83 | 0.97 | 0.78 | 0.83 | 0.97 | 0.85 | 0.83 |
|  | S669 | 1.53 | 0.45 | 0.73 | 1.56 | 0.44 | 0.72 | 1.55 | 0.59 | 0.72 |

***Table 6.* Performance comparison of sequence-based stability changes prediction methods on CAGI5 Challenge’s Frataxin.**

| Model | Direct | | | Reverse | | | Overall | | |
| --- | --- | --- | --- | --- | --- | --- | --- | --- | --- |
|  | RMSE | PCC | ACC | RMSE | PCC | ACC | RMSE | PCC | ACC |
| **MuFaDDG** | **3.49** | **0.17** | **0.75** | **3.37** | **0.25** | **0.88** | **3.43** | **0.67** | **0.81** |
| DDGemb | 3.27 | 0.65 | 0.50 | 3.17 | 0.76 | 0.88 | 3.22 | 0.86 | 0.69 |
| INPS-Seq | 3.33 | 0.73 | 0.75 | 3.44 | 0.72 | 0.63 | 3.39 | 0.84 | 0.69 |
| DDGun | 2.25 | 0.89 | 0.63 | 2.34 | 0.89 | 0.50 | 2.30 | 0.89 | 0.56 |
| THPLM | 3.55 | 0.67 | 0.75 | 3.55 | 0.72 | 0.63 | 3.55 | 0.80 | 0.69 |

# Supplementary Figures


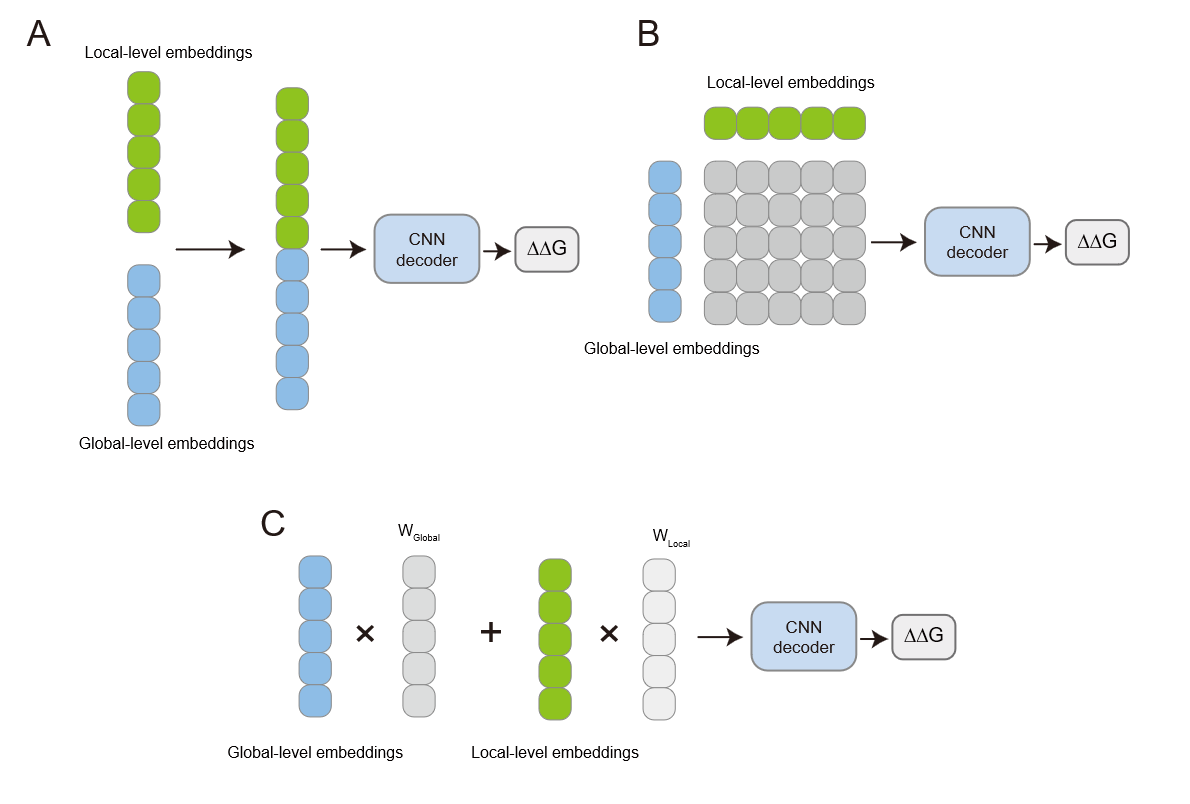


**Fig. 1.** Illustrates different feature integration methods: (A) the concatenation method, (B) the outer product method, and (C) the linear combination method.


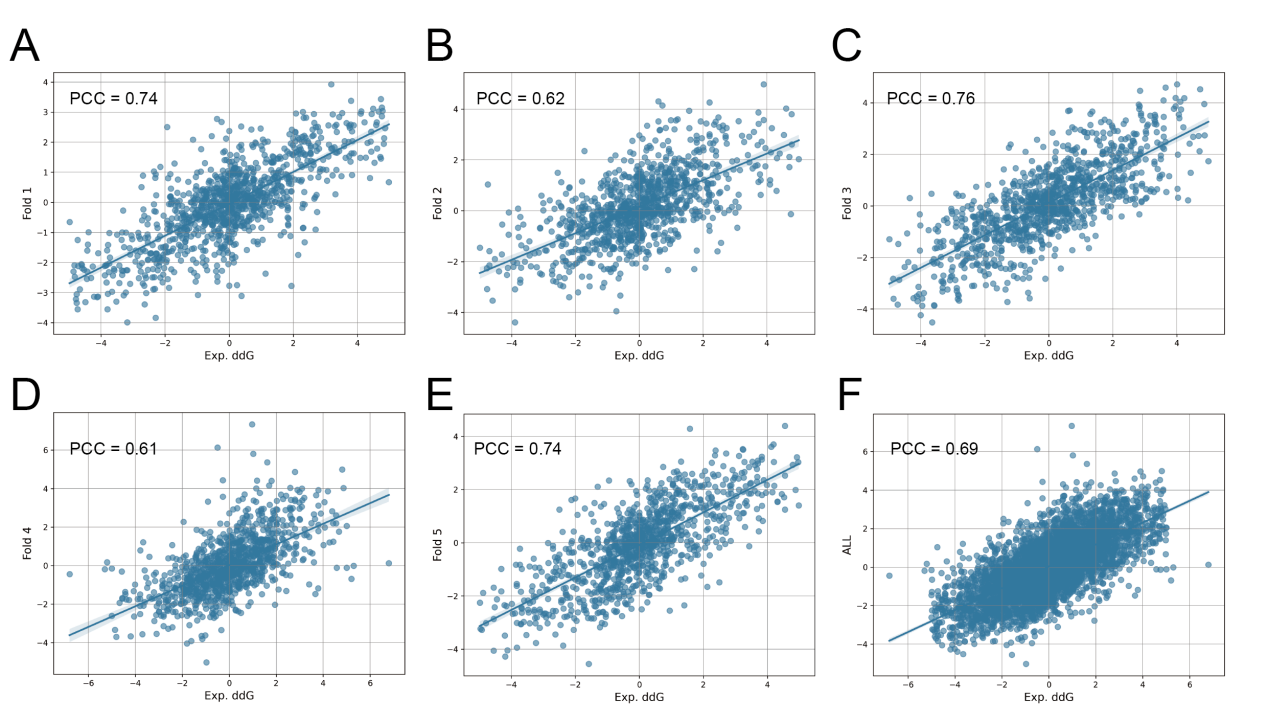


**Fig. 2.** Performance of the five cross-validation models on their corresponding validation sets(A-E) and the average performance on the S2648 dataset(F).


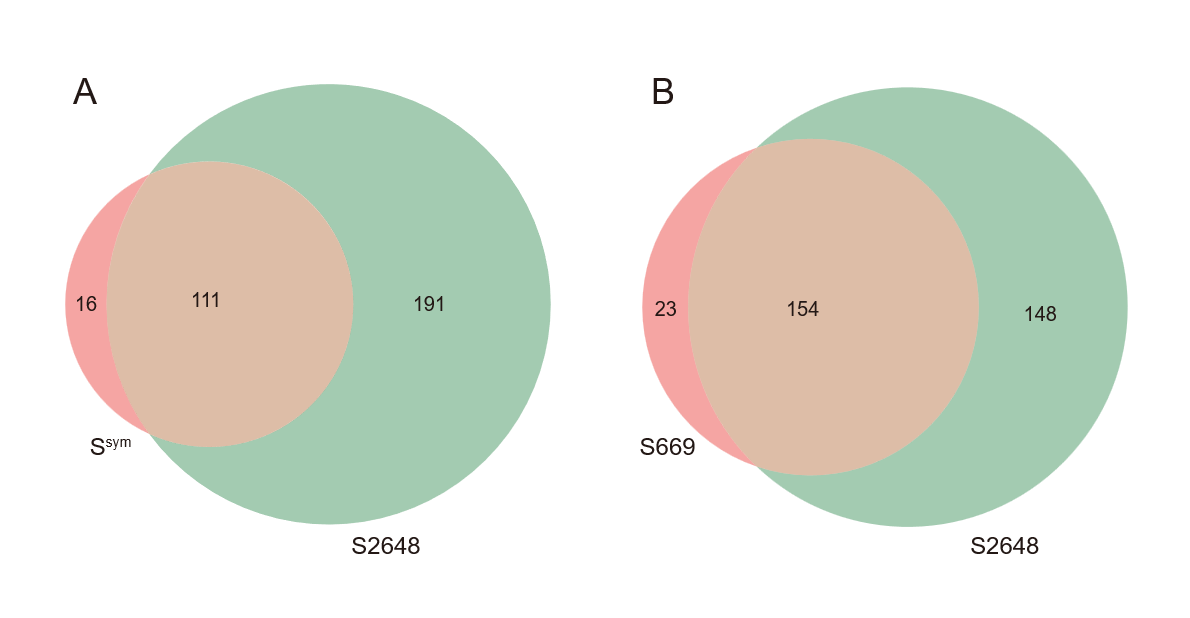


**Fig. 3.** Venn Diagrams of Mutation Combinations Between Ssym & S2648 and S669 & S2648. **(A)** Ssym Venn Diagram: Overlap of mutation combinations (WILD_RES → MUTANT_RES) between Ssym and S2648 datasets. The shared mutations appear in the intersection, while unique mutations are in non-overlapping areas.**(B)** S669 Venn Diagram: Overlap of mutation combinations between S669 and S2648 datasets, with shared and unique mutations similarly represented.


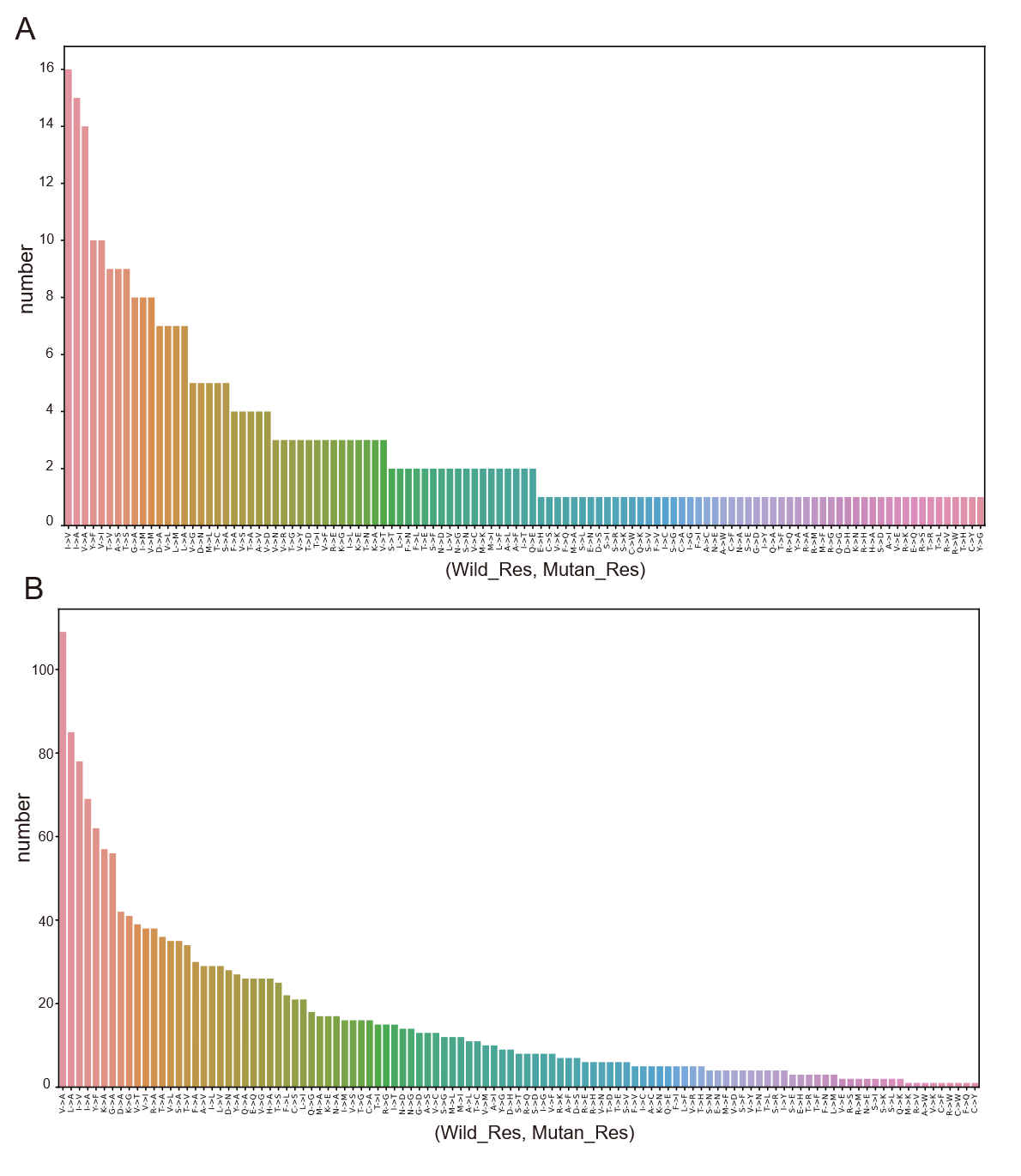


**Fig. 4.** Frequency of overlapping mutation combinations between the S2648 and Ssym datasets, shown for the Ssym dataset (A) and the S2648 dataset (B)

[1] Mishra S. K. PSP-GNM: Predicting Protein Stability Changes upon Point Mutations with a Gaussian Network Model[J]. Int J Mol Sci, 2022, 23(18).

[2] Worth C. L., Preissner R., Blundell T. L. SDM--a server for predicting effects of mutations on protein stability and malfunction[J]. Nucleic Acids Res, 2011, 39(Web Server issue): W215-222.

[3] Dehouck Y., Kwasigroch J. M., Gilis D.,et al. PoPMuSiC 2.1: a web server for the estimation of protein stability changes upon mutation and sequence optimality[J]. BMC Bioinformatics, 2011, 12: 151.

[4] Pires D. E., Ascher D. B., Blundell T. L. DUET: a server for predicting effects of mutations on protein stability using an integrated computational approach[J]. Nucleic Acids Res, 2014, 42(Web Server issue): W314-319.

[5] Pires D. E., Ascher D. B., Blundell T. L. mCSM: predicting the effects of mutations in proteins using graph-based signatures[J]. Bioinformatics, 2014, 30(3): 335-342.

[6] Masso M., Vaisman, II. AUTO-MUTE 2.0: A Portable Framework with Enhanced Capabilities for Predicting Protein Functional Consequences upon Mutation[J]. Adv Bioinformatics, 2014, 2014: 278385.

[7] Savojardo C., Fariselli P., Martelli P. L.,et al. INPS-MD: a web server to predict stability of protein variants from sequence and structure[J]. Bioinformatics, 2016, 32(16): 2542-2544.

[8] Laimer J., Hofer H., Fritz M.,et al. MAESTRO--multi agent stability prediction upon point mutations[J]. BMC Bioinformatics, 2015, 16: 116.

[9] Pucci F., Bernaerts K. V., Kwasigroch J. M.,et al. Quantification of biases in predictions of protein stability changes upon mutations[J]. Bioinformatics, 2018, 34(21): 3659-3665.

[10] Rodrigues C. H., Pires D. E., Ascher D. B. DynaMut: predicting the impact of mutations on protein conformation, flexibility and stability[J]. Nucleic Acids Res, 2018, 46(W1): W350-W355.

[11] Montanucci L., Capriotti E., Frank Y.,et al. DDGun: an untrained method for the prediction of protein stability changes upon single and multiple point variations[J]. BMC Bioinformatics, 2019, 20(Suppl 14): 335.

[12] Cao H., Wang J., He L.,et al. DeepDDG: Predicting the Stability Change of Protein Point Mutations Using Neural Networks[J]. J Chem Inf Model, 2019, 59(4): 1508-1514.

[13] Rodrigues C. H. M., Pires D. E. V., Ascher D. B. DynaMut2: Assessing changes in stability and flexibility upon single and multiple point missense mutations[J]. Protein Sci, 2021, 30(1): 60-69.

[14] Li B., Yang Y. T., Capra J. A.,et al. Predicting changes in protein thermodynamic stability upon point mutation with deep 3D convolutional neural networks[J]. PLoS Comput Biol, 2020, 16(11): e1008291.

[15] Chen Y., Lu H., Zhang N.,et al. PremPS: Predicting the impact of missense mutations on protein stability[J]. PLoS Comput Biol, 2020, 16(12): e1008543.

[16] Wang Shuyu, Tang Hongzhou, Zhao Yuliang,et al. BayeStab: Predicting effects of mutations on protein stability with uncertainty quantification[J]. Protein Sci, 2022, 31(11): e4467.

[17] Wang Shuyu, Tang Hongzhou, Shan Peng,et al. ProS-GNN: Predicting effects of mutations on protein stability using graph neural networks[J]. Computational Biology and Chemistry, 2023, 107: 107952.

[18] Dieckhaus H., Brocidiacono M., Randolph N. Z.,et al. Transfer learning to leverage larger datasets for improved prediction of protein stability changes[J]. Proc Natl Acad Sci U S A, 2024, 121(6): e2314853121.

[19] Diaz Daniel J., Gong Chengyue, Ouyang-Zhang Jeffrey,et al. Stability Oracle: a structure-based graph-transformer framework for identifying stabilizing mutations[J]. Nature Communications, 2024, 15(1): 6170.

[20] Cheng J., Randall A., Baldi P. Prediction of protein stability changes for single-site mutations using support vector machines[J]. Proteins, 2006, 62(4): 1125-1132.

[21] Huang L. T., Gromiha M. M., Ho S. Y. iPTREE-STAB: interpretable decision tree based method for predicting protein stability changes upon mutations[J]. Bioinformatics, 2007, 23(10): 1292-1293.

[22] Folkman L., Stantic B., Sattar A.,et al. EASE-MM: Sequence-Based Prediction of Mutation-Induced Stability Changes with Feature-Based Multiple Models[J]. J Mol Biol, 2016, 428(6): 1394-1405.

[23] Fariselli P., Martelli P. L., Savojardo C.,et al. INPS: predicting the impact of non-synonymous variations on protein stability from sequence[J]. Bioinformatics, 2015, 31(17): 2816-2821.

[24] Yang Y., Urolagin S., Niroula A.,et al. PON-tstab: Protein Variant Stability Predictor. Importance of Training Data Quality[J]. Int J Mol Sci, 2018, 19(4).

[25] Li G., Panday S. K., Alexov E. SAAFEC-SEQ: A Sequence-Based Method for Predicting the Effect of Single Point Mutations on Protein Thermodynamic Stability[J]. Int J Mol Sci, 2021, 22(2).

[26] Gong Jianting, Jiang Lili, Chen Yongbing,et al. THPLM: a sequence-based deep learning framework for protein stability changes prediction upon point variations using pretrained protein language model[J]. Bioinformatics, 2023, 39(11).

[27] Savojardo Castrense, Manfredi Matteo, Martelli Pier Luigi,et al. DDGemb: predicting protein stability change upon single-and multi-point variations with embeddings and deep learning[J]. Bioinformatics, 2025, 41(1): btaf019.

[28] Capriotti E., Fariselli P., Casadio R. I-Mutant2.0: predicting stability changes upon mutation from the protein sequence or structure[J]. Nucleic Acids Res, 2005, 33(Web Server issue): W306-310.

[29] Chen C. W., Lin J., Chu Y. W. iStable: off-the-shelf predictor integration for predicting protein stability changes[J]. BMC Bioinformatics, 2013, 14 Suppl 2: S5.

[30] Witvliet D. K., Strokach A., Giraldo-Forero A. F.,et al. ELASPIC web-server: proteome-wide structure-based prediction of mutation effects on protein stability and binding affinity[J]. Bioinformatics, 2016, 32(10): 1589-1591.

[31] Quan L., Lv Q., Zhang Y. STRUM: structure-based prediction of protein stability changes upon single-point mutation[J]. Bioinformatics, 2016, 32(19): 2936-2946.

[32] Pancotti C., Benevenuta S., Repetto V.,et al. A Deep-Learning Sequence-Based Method to Predict Protein Stability Changes Upon Genetic Variations[J]. Genes-Basel, 2021, 12(6).

[33] Chen C. W., Lin M. H., Liao C. C.,et al. iStable 2.0: Predicting protein thermal stability changes by integrating various characteristic modules[J]. Comput Struct Biotechnol J, 2020, 18: 622-630.
